# Effects of plyometric and whole-body vibration on physical performance in collegiate basketball players: a crossover randomized trial

**DOI:** 10.1038/s41598-022-09142-8

**Published:** 2022-03-23

**Authors:** Pratyakshi Munshi, Moazzam Hussain Khan, Nitin Kumar Arora, Shibili Nuhmani, Shahnawaz Anwer, Heng Li, Ahmad H. Alghadir

**Affiliations:** 1 Centre for Physiotherapy and Rehabilitation Sciences, Jamia Millia Islamia, New Delhi, India; 2 Hochschule für Gesundheit, 44801 Bochum, Germany; 3 Department of Physical Therapy, College of Applied Medical Sciences, Imam Abdulrahman Bin Faisal University, Dammam, Saudi Arabia; 4 Rehabilitation Research Chair, College of Applied Medical Sciences, King Saud University, Riyadh, Saudi Arabia; 5 Department of Building and Real Estate, Faculty of Construction and Environment, Hong Kong Polytechnic University, Kowloon, Hong Kong Special Administrative Region China

**Keywords:** Health care, Medical research

## Abstract

While many studies suggested the isolated effects of plyometric and whole-body vibration exercises on physical performance variables, only few studies have compared the acute effects of plyometric and whole-body vibration on the occurrence of post-activation potentiation and the resultant improvements in performance. Therefore, we aimed to compare the acute effects of plyometric exercises and whole-body vibration training on physical performance in collegiate basketball players. Twenty-four collegiate male basketball players (age 20.8 ± 2.02 years, height 1.79 ± 0.7 m, and weight 71.2 ± 7.6 kg) participated in this randomized crossover study. Subjects were received both plyometric and whole-body vibration exercises after a 48-h washed-out period. Countermovement Jump height, sprint, and agility time were measured at baseline, 4- and 12-min post-plyometric, and whole-body vibration exercises. The result suggests a positive effect of both the plyometric and whole-body vibration exercises on countermovement jump and agility time (*p* = 0.001). While the countermovement jump height and agility were higher in the plyometric group (mean difference 1.60 cm and 0.16 s, respectively), the sprint performance was higher in the whole-body vibration group. However, these differences were statistically non-significant between the two groups (*p* > 0.05). This study suggests that both plyometric and whole-body vibration exercises may improve post-activation potentiation, which leads to better physical performance.

Trial registration CTRI/2019/05/019059. Registered with the Clinical trials registry, India on 10/05/2019. http://ctri.nic.in/Clinicaltrials/advsearch.php.

## Introduction

Warm-up helps improve the optimum force, maximum peak acceleration, and rate of force development by increasing the recruitment of motor units, firing the muscle spindles, and increasing synergistic musculature. Additionally, it also aids in reducing the inhibition of the Golgi apparatus and psychological effects; all of which together directly or indirectly influence post-activation potentiation^1^. Therefore, warm-ups eliciting post-activation potentiation may be the key to improved power performance.

Post-activation potentiation (PAP) is the process in which muscle performances are acutely enhanced due to their contractile property^2,3^. There is considerable literature in favor of using conditioning activity (performance of maximum or near-maximum muscle contraction) to stimulate enhancement in subsequent upper-body ballistic performance, jumping, sprinting, and throwing^2,4^. Previous studies have enumerated various contributory mechanisms following PAP^2,5–7^. For example, phosphorylation of light chains controlling myosin is one way by which the protein filaments actin and myosin become sensitive to calcium (Ca2+) release^2,5^, while another is an increase in the recruitment of higher-order motor units^2,6,7^. Past studies demonstrated that subject features such as training condition (strength levels) and type of fiber distribution may determine the ability to display PAP^8^. Last few decades, researchers tried to examine the effects of strategies like PAP on athletic performance using dynamic movements such as plyometrics, back squats, resistance training^9^, whole-body vibration (WBV)^10–12^, sled towing^13^, and isometric maximum voluntary contractions^13,14^. A previous study suggested that an increase in countermovement jump (CMJ) height and maximum force is due to induced PAP after 1–5 min of plyometric exercises^15^. Another study reported an increased CMJ power by about 2% after the completion of five modified drop jumps at 1 min of rest-interval^1^. Recently, Zagatto et al.^16^ suggested that the improvements in performance variables following exercise interventions might be attributed to the co-existence of PAPE (post-activation performance enhancement) along with PAP.

Likewise, plyometric exercise has also been a cornerstone as a strategy to improve power and strength performance in athletic population^17^. This training strategy utilizes a stretch–shortening cycle that involves an eccentric stretch to the muscle followed by an immediate concentric contraction. It helps in improving the reaction time by maximizing the force generation in the muscle tendon unit^18^. Plyometric training has previously been reported as a means to enhance the jumping ability^19^ and reducing sprint timings^20^. WBV is an alternative exercise method used to improve muscle power^21,22^, strength^23,24^, and flexibility^23^. WBV is implemented on a platform that typically vibrates between 30 and 50 Hz by standing, squatting, or performing dynamic movements. Physiologically, WBV is proposed to activate α-motor neurons to improve muscle performance by increasing muscle activation, stretch reflex potentiation, antagonistic muscle inhibition, and synchronization of the motor unit^25,26^.

Cochrane et al.^11^ investigated the effect of WBV (36 Hz) and 5 min of static bodyweight squat on post-activation potentiation, muscle twitch, and patellar reflex properties among 12 national-level athletes. They found an increased muscle peak force of about 12% and a force production rate of about 11% following a WBV exercise. Likewise, Ronnestad et al.^27^ and Padulo et al.^28^ used WBV exercises to improve 40 m sprint (~ 0.65%) and repeated sprint performance (~ 4%) in soccer players. Additionally, Haris et al.^29^ and Pojskic et al.^30^ reported that WBV with the addition of 30% of body weight may increase CMJ height (~ 5.5%), and decreased sprint and agility time (1.9%).

Thus, many studies have suggested the effects of isolated plyometric and WBV on PAP^27–30^, only few studies have compared the acute effects of plyometric and WBV on the occurrence of post-activation potentiation^11,31^. To the best of our knowledge, no study compared the acute effects of plyometrics and WBV on CMJ height, sprint, and agility. We hypothesize that acute WBV exercises will result in significant gains in CMJ height, sprint speed, and agility in male basketball players when compared to acute plyometric exercises. Additionally, the WBV training protocol requires less time than the plyometric routine. Thus, if acute WBV is found to be superior, it may aid in achieving more rapid gains in performance metrics with short-term exercise programs. Therefore, this study compared the acute effects of plyometrics and WBV on PAP in collegiate basketball players by measuring physical performance.

## Materials and methods

### Participants

Twenty-four university basketball players (age 20.8 ± 2.02 years, height 1.79 ± 0.7 m, weight 71.2 ± 7.6 kg, and body mass index 22.00 ± 1.49 kg/m^2^) participated in this randomized crossover study. The sample size was determined using software G*Power Version 3.1.9.2^32^ using the data of a previous study done by Dallas et al.^33^, in which change in CMJ performance was analyzed and 24 subjects (considering 12% dropout) with an effect size of 0.34, an alpha level of 0.05 and power (1-beta) of 0.80 was calculated. The subjects included in this study performed resistance training 3 days per week and were not accustomed to WBV training. Participants were included if they were between the age group of 18–25 years, a member of a collegiate male basketball team, continuously playing for more than 2 years at the university level, involved in sport-specific training for at least 2 days per week, and playing competitive sports once a week. Participants were excluded if they had a history of any surgery or injury of the lower extremities in the past 1-year, joint instability, musculo-tendinous injury, or musculoskeletal disorders that would prohibit the subject to participate in sports and who were taking performance-enhancing supplements^34^. All testing and training were performed at the sports ground, Jamia Millia Islamia, New Delhi.

### Ethical considerations

The study procedure was approved by the institutional ethics committee of Jamia Millia Islamia, New Delhi (No. 31/10/188/JMI/IE/2018). The subjects provided written informed consent. All work was conducted in accordance with the principles and procedures outlined in the Helsinki Declaration. The clinical trial protocol was registered with the Clinical Trials Registry of India (CTRI/2019/05/019059; date of registration: 10/05/2019) and was made available to the public.

### Randomization and crossover

**Figure 1:**
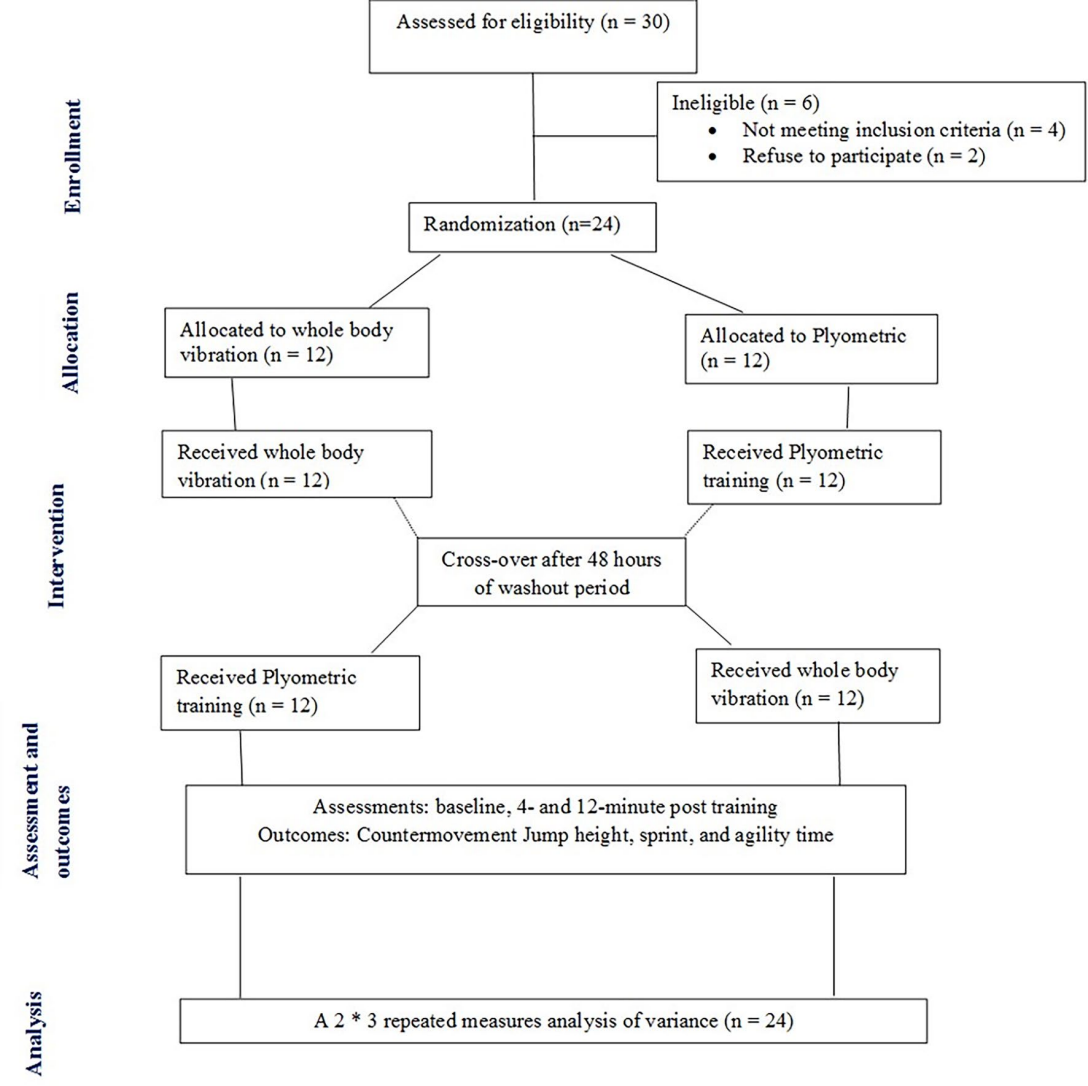
Flow diagram of participants through each stage of the randomized trial.

The participants were randomly assigned to WBV or Plyometric training. Blank folders were numbered from 1 to 24, given concealed codes for group assignment by an independent assessor, and kept in a safe locker. Once a participant fulfilled the eligibility criteria and agreed to participate, an independent assessor drew the next folder of the file to assign the group. Participants were randomized to first receive either WBV or plyometric training and after 48 h of the wash-out period^35^, they were crossed over to receive the opposite intervention. Participants in group one (n = 12) first did WBV followed by plyometric training, while the other group (n = 12) first did plyometric training followed by WBV (Fig. 1).

### Testing

Participants were screened to rule out any disease with the help of a medical screening questionnaire and they were given 1 day rest from the game before the training and testing to avoid the training effects. Participants were instructed not to perform any form of exercise and not to consume alcohol or coffee 24 h prior to each session^36^, as it may affect the training and testing^37^. A familiarization session was held for both the testing and training methods.

Before the training, general warm-up exercises were performed in both the groups and included 5 min of submaximal jogging (4.3–7.5 mph on a treadmill) and active-static stretching of the major muscles of the lower limb (2 repetitions of gluteus, quadriceps, hamstrings, and calf stretching for 30 s each). After 1-min rest, the three trials of baseline measurements of CMJ, sprint, and agility performance were obtained and the best of three trials were recorded. After 5 min, they were asked to receive either the plyometric training or WBV as per group allocation. Three trials of posttest measurements of CMJ, height, sprint time and agility were taken at 4- and 12-min after training and the best of the three trials were recorded. Participants were asked to take a 48-h rest to minimize the fatigue effects on test performance.

### Training

Plyometric training protocol^38^ included double-legged vertical (5 sets of 10 repetitions each) and broad jumps (2 repetitions of 15 m distance), single and double legged bounding (single repetition of 30 m distance) and depth jumps (single set of 5 repetitions), all were completed from a height of 40 cm for a duration of 30 s each. Participants were asked to minimize ground contact during bounding depth jump exercises and asked to achieve a maximal height during the exercises. A 15–30 s of recovery time between repetition and sets was given.

In WBV training^33^, participants were asked to stand on a WBV platform in which they were exposed to a vertical sinusoidal mechanical WBV. A 30 Hz vibration frequency and 5 mm amplitude of WBV dose was given for 2 min. Participants were given a single bout of WBV training during two 30-s squatting exercise sets and two 30-s single-leg squatting exercise sets with 30-s rest intervals.

### Outcomes

#### CMJ height

The CMJ test was used to find the strength of the lower limbs^39^. Participants applied ink at the end of their fingertips using a stamp pad. The participants were instructed to stand aside 15 cm from the marking board, keeping both feet remaining on the ground. They asked to reach up as high as possible with one hand and marked onto the marking board with the fingertip. This is the standing reach height. The participants were then instructed to jump vertically (90° knee bend) as maximum as can while actively swinging the arms and marking on the board. The height of the jump was determined using a measuring tape attached to a graph paper that marked the initial and final jump ink prints of each participant. With an ICC value of 0.98, CMJ height has been demonstrated to possess a high reliability^40^.

#### 20-m single sprint

20-m sprint was used to assess the speed performance^41^. Two cones were placed 20 m apart. Participants ran on a call of ready-get set-go and were asked to complete the 20 m sprint as quickly as possible. The timing was recorded with the digital stopwatch in seconds. A 20-m sprint test showed a high level of intra-rater reliability in healthy male participants (r = 0.91) and no prior practice session was required^42^. This test was also positively correlated to playing duration in male basketball players (r = 20.62)^43^.

#### Agility T-test

**Figure 2:**
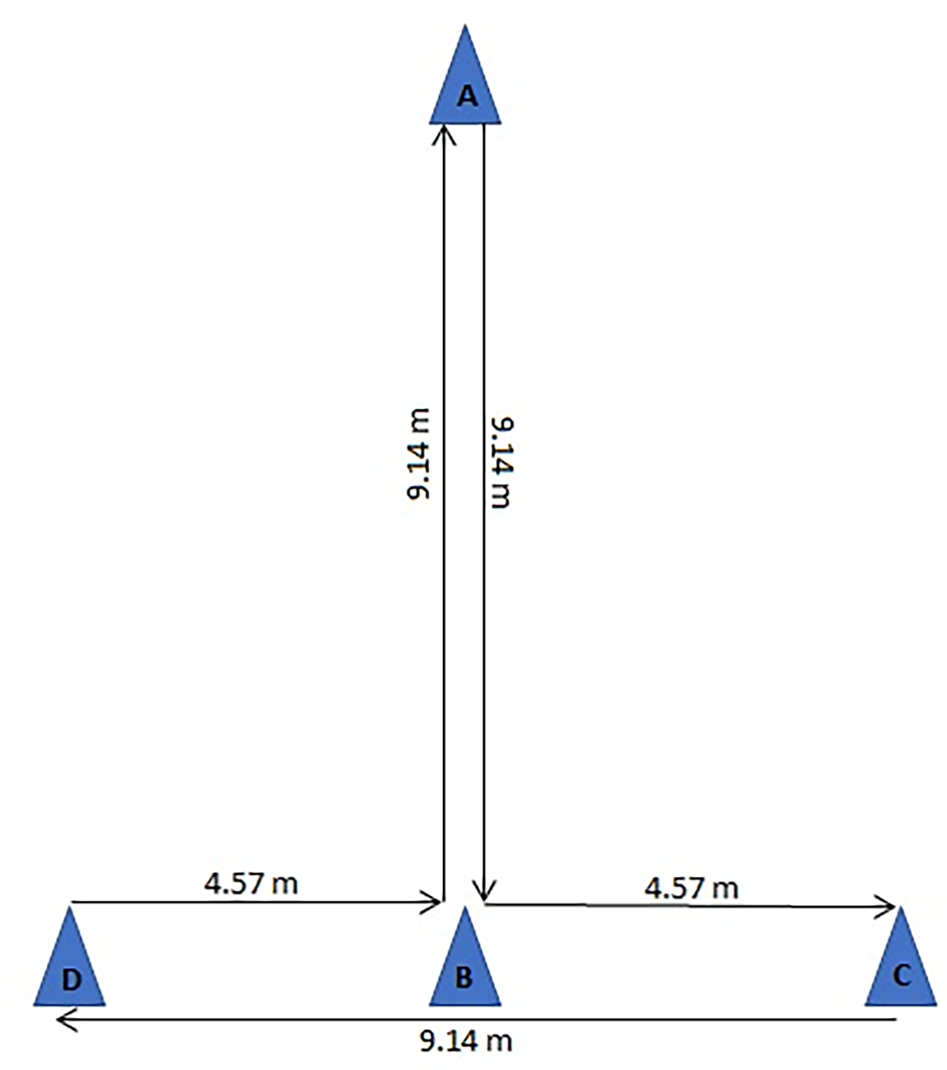
Diagrammatic representation of agility T test.

Four cones were placed at a distance of 4.57 m and 9.14 m in a T shape. The participants were asked to start at cone A. On the command of ready–get–set–go, to run touched cone B and shuffled sideways to the left and touched cone C. Then shuffled sideways to the right and touched cone D. Finally, they shuffled back to the left and touched cone B, and return to cone A. Once they crossed cone A, the stopwatch was stopped^29^ (Fig. 2). A high intra-class reliability of agility T test has been shown previously^44^.

### Statistical analysis

Statistical analysis was done using SPSS software 21.0 (SPSS Inc. Chicago, IL, USA). The descriptive data are presented as mean ± standard deviation. Shapiro–Wilk test was used to confirm the normality of the distribution scores. A 2 × 3 repeated measures analysis of variance (ANOVA) with time (at baseline, 4 min, 12 min of posttest), protocol (plyometric training and WBV), and the interaction effect (time × protocol) was used. If the main effect of the protocol was not significant, post hoc analysis was not employed. Whereas, if the main effect of time was significant, a post hoc analysis using Bonferroni correction was applied on time. The significance level was set at *p* < 0.05.

## Results

**Figure 3:**
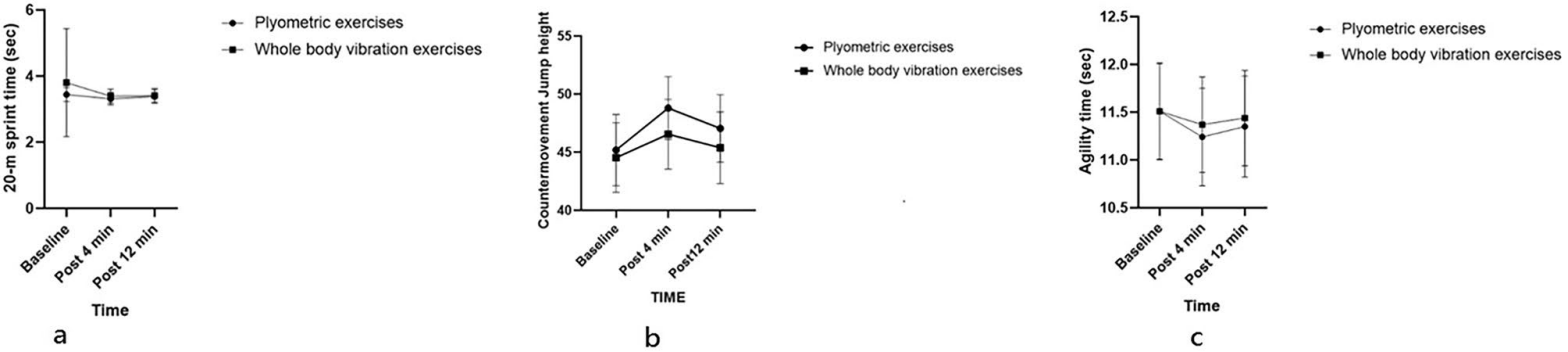
(**a**) Maximal height in the Counter movement Jump performance for plyometrics and whole-body vibration group at specified intervals; (**b**) Maximal 20-m sprint time for the plyometric and whole-body vibration group at specified time intervals; (**c**) Maximal agility time for the plyometric and whole-body vibration group at specified time intervals**.**

Descriptive statistics of dependent variables are presented in Table 1. CMJ height had a significant effect with respect to time (*p* = 0.001), the effect of the protocol was non-significant (*p* = 0.807), and the time × protocol interaction effect was also significant (*p* = 0.001), indicating that CMJ improved following both protocols and there was an insignificant difference between the plyometric and WBV exercises (Table 2). A post hoc pairwise comparison with respect to time showed a significant increase in height from the baseline to the 4th minute (*p* = 0.001) and from baseline to the 12th minute (*p* = 0.001) (Table 3, Fig. 3a).

**Table 1:** Descriptive statistics of dependent variables.

Dependent variable	Time (min)	Plyometric exercise	WBV exercises
CMJ (cm)	Baseline	45.18 ± 3.06	44.53 ± 2.99
	Post 4 min	48.80 ± 2.70	46.55 ± 3.00
	Post 12 min	47.05 ± 2.91	45.38 ± 3.07
Sprint (s)	Baseline	3.44 ± 0.21	3.80 ± 1.64
	Post 4 min	3.31 ± 0.19	3.39 ± 0.21
	Post 12 min	3.38 ± 0.20	3.41 ± 0.21
Agility (s)	Baseline	11.51 ± 0.51	11.51 ± 0.50
	Post 4 min	11.24 ± 0.51	11.37 ± 0.50
	Post 12 min	11.35 ± 0.53	11.44 ± 0.50

N = 24; *CMJ* countermovement jump, *min* minute, *SD* standard deviation.

**Table 2:** Two-way (2 × 3) repeated measures analysis of variance.

Variable	Source	Df	Partial ŋ^2^	F-value	*p* value
CMJ	Time	1.469	0.530	24.829	0.001*
	Protocol	1	0.003	0.061	0.807
	Time × protocol	1.357	0.874	152.281	0.001*
Sprint	Time	1.010	0.058	1.359	0.267
	Protocol	1.000	0.042	0.964	0.337
	Time × protocol	1.004	0.089	2.154	0.156
Agility	Time	1.866	0.580	30.413	0.001*
	Protocol	1.000	0.099	2.405	0.135
	Time × protocol	1.162	0.819	99.681	0.001*

N = 24; *CMJ* Countermovement Jump.

*Significant differences at *p* < 0.01.

**Table 3:** Post hoc pairwise comparison with time.

Variables	T_1_ versus T_2_	T_2_ versus T_3_	T_1_ versus T_3_
CMJ	0.001*	0.673	0.001*
Sprint	0.646	0.958	0.015*
Agility	0.001*	0.002*	0.001*

N = 24; *CMJ* Countermovement Jump, *T*_*1*_ at baseline, *T*_*2*_ at 4-min, *T*_*3*_ at 12-min.

*Significant difference at *p* < 0.01.

20 m Sprint had a non-significant effect with respect to time (*p* = 0.267), protocol (*p* = 0.337), and the time × protocol interaction was also non-significant (*p* = 0.156) (Table 2, Fig. 3b).

Agility time had a significant effect with respect to time (*p* = 0.001) and the time × protocol interaction effect (*p* = 0.001), however, the protocol was non-significant (*p* = 0.135), indicating that agility was improved following both protocols and there was a statistically insignificant difference between the plyometric and WBV exercises (Table 2). Post hoc pairwise comparison for time showed a significant decrease in agility time from baseline to the 4th minute (*p* = 0.001), from the 4th minute to the 12th minute (*p* = 0.002), and from baseline to the 12th minute (*p* = 0.001) (Table 3 Fig. 3c).

## Discussion

The result of this study shows an acute positive impact of both plyometric and WBV exercises on CMJ and agility. While the CMJ height and agility were improved more with plyometric exercise and sprint performance improved more with WBV exercise, there was a statistically insignificant difference between the two protocols. As compared to the baseline, CMJ height increased by 8.01% and 4.53% after 4 min of plyometric exercise and WBV, respectively. However, after 12 min of plyometric and WBV exercises, the CMJ height increased by 4.13% and 1.90%, respectively. The enhancement in the protocols can be speculated by an increase in the neuromuscular responses. In a previous study, Tobin et al.^45^ reported increased CMJ heights by 4.8%, 3.9%, and 3.5% after 1, 3, and 5 min of plyometric exercise, respectively. Similarly, Requena et al.^46^ reported an increase in CMJ height of 3.08 cm at a 5-min rest interval after 25 plyometric repetitions. Additionally, Sharma et al.^35^ reported decreased CMJ height by 4.8% immediately after plyometric exercise, however, after 10 min of recovery the CMJ height was increased by 13%. On the contrary, Esformes et al.^47^ reported no additional benefit of plyometric exercise in increasing the performance of CMJ height. However, the protocol they used was a single 70-s plyometric exercise effort. The long duration of the effort must have led to an increased level of metabolic fatigue that interfered with the response to potentiation. Similarly, Till et al.^48^ observed no additional benefits of plyometric exercise on CMJ's performance.

In line with the current results, Dallas et al.^33^ showed an increase in CMJ performance of 6.51% and 4.57% at 1 min and 15 min after WBV exercise. The protocol used in this study was like our study. Likewise, Wu et al.^49^ also showed acute WBV to be effective in producing significant improvements in agility and speed in male volleyball players following 1 min WBV exposure at 30 Hz. Similarly, Naclerio et al.^50^, observed an enhanced CMJ performance after a 4-min post-WBV exercise. Additionally, Cormie et al.^51^ reported an increased CMJ performance after 5- and 10-min post-WBV exercises. In contrast, while Rittweger et al.^52^ found a reduced CMJ performance by 9.1% after WBV exercise, other studies reported no changes in CMJ performance after WBV exercise^53,54^.

With both protocols, our study showed no significant improvement in the 20 m sprint. However, the average sprint time was reduced by 3.77% and 10.78% in 4-min post-plyometric and WBV exercises, respectively. Similarly, the average sprint time was reduced by 1.74% and 10.26% in 12-min post-plyometric and WBV exercises. These results indicate that the sprint time was reduced more after WBV than that of plyometric exercise. Many past studies have shown the interactions between post-activation potentiation and sprint performance. For example, Turner et al.^55^ found improved sprint performance by 1.9% in 4-min and 2.3% in 8-min post-plyometric exercises. The speculated mechanism for this potentiation was enhanced activation of the musculature and increased recruitment of type 2 motor units^55^. However, these improvements are greater than the minimal worthwhile change of < 0.01 s^56^. Sharma et al.^35^ reported increased sprint time by 2.4% immediately after plyometrics, however, the sprint time was reduced by 8.9% after 10-min of recovery. This improvement in sprint performance could be because of optimal motor neuron excitability and recruitment of fast-twitch fibers^57^. Pojskic et al.^30^ observed an improvement in sprint performance after 2-min of recovery following WBV exercise. In contrast, Bullock et al.^53^ and Kavanaugh et al.^58^ reported no benefit of using WBV exercises to elicit potentiation in sprint performance. The reason for this could be that the intensity of the exercise used was not enough to produce any enhancement or potentiation.

Our study showed that compared to baseline, the agility time was reduced by 2.34% and 1.21% in 4-min post-plyometric and WBV exercises, respectively. However, the agility time was reduced by 1.39% and 0.60% after 12-min post-plyometric and WBV exercises. Agility performance was improved in both protocols; however, it was more enhanced with the plyometric protocol. Only a few studies have shown the interactions between post-activation potentiation and agility performance. Consistent with the current results, previous studies have shown that sufficient recovery time is required to reduce fatigue and carry out PAP^59,60^. Agility time in our study showed an improvement which supports the finding of Young et al.^59^ and other researchers^61,62^, as they also documented the relationship between agility and post-activation potentiation phenomenon and explained the neural activation of the phenomenon. Only a few researches have investigated the effect of WBV exercise on agility performance. For example, Pojskic et al.^30^ observed an enhanced improvement in agility performance after WBV exercise. Similarly, Pienaar et al.^63^ reported an improvement in agility time after WBV exercise. In contrast, Cochrane et al.^12^ and Torvinen et al.^64^ observed no significant enhancement in agility after WBV exercise. It can be speculated that the volume of the stimulus was not enough to enhance the acute performance.

### Limitations

This study acknowledged some potential limitations. First, a stopwatch was used to measure the timing of agility and sprint, however, it is not considered a reliable and accurate method. Consequently, to reduce chances of errors, the tester was trained multiple times prior to the testing procedure and the same person measured the time on every testing session. Therefore, an advanced method such as timing gates may be used to measure more accurate values in future studies. Second, PAP was not recorded with the help of electromyography. Therefore, future studies can be performed to measure and compare muscular activity and potentiation by using electromyography after plyometric and WBV exercises. Third, while participants were asked not to consume alcohol or coffee prior to testing, their eating habits were not monitored. Fourth, individual depth jump heights were not determined, which may have impacted the effects of the activity on subjects of varying heights. Fifth, this study is limited to collegiate male basketball players, and therefore, the results cannot be generalized to the whole population.

## Conclusion

This study indicates that neither plyometric nor WBV exercises provide an additional benefit when compared to plyometric training for improving countermovement jump and agility performance in male basketball players. As a result, additional equipment is not required, and plyometric exercises alone can serve as an appropriate modality for improving the fitness characteristics examined. If logistics allowed, athletes could alternate between plyometric and WBV exercises during their periodized training routines or warmup/cooldown phases.

## Data Availability

All data generated or analyzed during this study are presented in the manuscript. Please contact the corresponding author for access to data presented in this study.

## References

[1] Hilfiker R, Hübner K, Lorenz T, Marti B (2007). Effects of drop jumps added to the warm-up of elite sport athletes with a high capacity for explosive force development. J. Strength Cond. Res..

[2] Robbins DW (2005). Postactivation potentiation and its practical applicability: A brief review. J. Strength Cond. Rese..

[3] Tseng K-W, Chen J-R, Chow J-J, Tseng W-C, Condello G, Tai H-L (2021). Post-activation performance enhancement after a bout of accentuated eccentric loading in collegiate male volleyball players. Int. J. Environ. Res. Public Health.

[4] Tillin NA, Bishop D (2009). Factors modulating post-activation potentiation and its effect on performance of subsequent explosive activities. Sports Med..

[5] Seitz LB, Haff GG (2016). Factors modulating post-activation potentiation of jump, sprint, throw, and upper-body ballistic performances: A systematic review with meta-analysis. Sports Med..

[6] Maffiuletti NA, Aagaard P, Blazevich AJ, Folland J, Tillin N, Duchateau J (2016). Rate of force development: Physiological and methodological considerations. Eur. J. Appl. Physiol..

[7] Blazevich AJ, Babault N (2019). Post-activation potentiation versus post-activation performance enhancement in humans: Historical perspective, underlying mechanisms, and current issues. Front. Physiol..

[8] Hamada T, Sale DG, MacDougall JD, Tarnopolsky MA (2000). Postactivation potentiation, fiber type, and twitch contraction time in human knee extensor muscles. J. Appl. Physiol..

[9] Bazett-Jones DM, Winchester JB, McBride JM (2005). Effect of potentiation and stretching on maximal force, rate of force development, and range of motion. J. Strength Cond. Res..

[10] Cochrane DJ, Stannard SR (2005). Acute whole body vibration training increases vertical jump and flexibility performance in elite female field hockey players. Br. J. Sports Med..

[11] Cochrane DJ, Stannard SR, Firth EC, Rittweger J (2010). Acute whole-body vibration elicits post-activation potentiation. Eur. J. Appl. Physiol..

[12] Cochrane DJ, Booker H (2014). Does acute vibration exercise enhance horizontal jump performance?. J. Sports Sci. Med..

[13] Wong M, Dobbs I, Watkins C, Barillas S, Lin A, Archer D (2017). Sled towing acutely decreases acceleration sprint time. J. Strength Cond. Res..

[14] French DN, Kraemer WJ, Cooke CB (2003). Changes in dynamic exercise performance following a sequence of preconditioning isometric muscle actions. J. Strength Cond. Res..

[15] Gossen ER, Sale DG (2000). Effect of postactivation potentiation on dynamic knee extension performance. Eur. J. Appl. Physiol..

[16] Zagatto AM, Claus GM, Dutra YM, de Poli RA, Lopes VHF, Goodall S (2022). Drop jumps versus sled towing and their effects on repeated sprint ability in young basketball players. BMC Sports Sci. Med. Rehabil..

[17] Chu DC (1983). Plyometrics: The link between strength and speed. Nat. Strength Cond. Assoc. J..

[18] Rassier DE, Herzog W (2005). Force enhancement and relaxation rates after stretch of activated muscle fibres. Proc. R. Soc. B Biol. Sci..

[19] Chimera NJ, Swanik KA, Swanik CB, Straub SJ (2004). Effects of plyometric training on muscle-activation strategies and performance in female athletes. J. Athl. Train..

[20] Rimmer E, Sleivert G (2000). Effects of a plyometrics intervention program on sprint performance. J. Strength Cond. Res..

[21] Chen CH, Liu C, Chuang LR, Chung PH, Shiang TY (2014). Chronic effects of whole-body vibration on jumping performance and body balance using different frequencies and amplitudes with identical acceleration load. J. Sci. Med. Sport.

[22] Lamont HS, Cramer JT, Bemben DA, Shehab RL, Anderson MA, Bemben MG (2010). The acute effect of whole-body low-frequency vibration on countermovement vertical jump performance in college-aged men. J. Strength Cond. Res..

[23] Karatrantou K, Gerodimos V, Dipla K, Zafeiridis A (2013). Whole-body vibration training improves flexibility, strength profile of knee flexors, and hamstrings-to-quadriceps strength ratio in females. J. Sci. Med. Sport.

[24] Lovell R, Midgley A, Barrett S, Carter D, Small K (2013). Effects of different half-time strategies on second half soccer-specific speed, power and dynamic strength. Scand. J. Med. Sci. Sports.

[25] Cardinale M, Bosco C (2003). The use of vibration as an exercise intervention. Exer. Sport Sci. Rev..

[26] Sharma S, Saifi S, Krarora N, Sharma S (2021). Whole body vibration for athletes: An evidence informed review. J. Clin. Diagn. Res..

[27] Rønnestad BR, Ellefsen S (2011). The effects of adding different whole-body vibration frequencies to preconditioning exercise on subsequent sprint performance. J. Strength Cond. Res..

[28] Padulo J, Di Giminiani R, Ibba G, Zarrouk N, Moalla W, Attene G (2014). The acute effect of whole body vibration on repeated shuttle-running in young soccer players. Int. J. Sports Med..

[29] Haris MH, Khan MH, Tansswar T, Irshad N, Nuhmani S (2021). Acute effects of weighted plyometric exercise on sprint, agility and jump performance in university football players. Phys. Act. Rev..

[30] Pojskić H, Pagaduan JC, Babajić F, Užičanin E, Muratović M, Tomljanović M (2015). Acute effects of prolonged intermittent low-intensity isometric warm-up schemes on jump, sprint, and agility performance in collegiate soccer players. Biol. Sport.

[31] Chen ZR, Lo SL, Wang MH, Yu CF, Te Pend H (2017). Can different complex training improve the individual phenomenon of post-activation potentiation?. J. Hum. Kinet..

[32] Erdfelder E, Faul F, Buchner A (1996). GPOWER: a general power analysis program. Behav. Res. Methods Instrum. Comput..

[33] Dallas G, Kirialanis P, Mellos V (2014). The acute effect of whole body vibration training on flexibility and explosive strength of young gymnasts. Biol. Sport.

[34] Peeling P, Binnie MJ, Goods PSR, Sim M, Burke LM (2018). Evidence-based supplements for the enhancement of athletic performance. Int. J. Sport Nutr. Exer. Metab..

[35] Sharma SK, Raza S, Moiz JA, Verma S, Naqvi IH, Anwer S (2018). Postactivation potentiation following acute bouts of plyometric versus heavy-resistance exercise in collegiate soccer players. BioMed Res. Int..

[36] Watt K, Purdie DM, Roche AM, McClure RJ (2005). The relationship between acute alcohol consumption and consequent injury type. Alcohol Alcohol..

[37] Astorino TA, Roberson DW (2010). Efficacy of acute caffeine ingestion for short-term high-intensity exercise performance: A systematic review. J. Strength Cond. Res..

[38] Luebbers PE, Hulver MW, Thyfault JP, Carper MJ, Lockwood RH, Potteiger JA (2003). Effects of plyometric training and recovery on vertical jump performance and anaerobic power. Med. Sci. Sports Exer..

[39] Roschel H, Batista M, Monteiro R, Bertuzzi RC, Barroso R, Loturco I (2009). Association between neuromuscular tests and kumite performance on the Brazilian Karate National Team. J. Sports Sci. Med..

[40] Markovic G, Dizdar D, Jukic I, Cardinale M (2004). Reliability and factorial validity of squat and countermovement jump tests. J. Strength Cond. Res..

[41] Khan MH, Nuhmani S, Kapoor G, Ahmad N, Agnihotri D (2012). Effects of ice with active warmup and active warmup alone on performance in football player. Int. J. Biomed. Adv. Res..

[42] Moir G, Glaister M (2004). The reliability of accelerative sprint performance: Does starting position matter?. J. Hum. Mov. Stud..

[43] Hoffman JR, Tenenbaum G, Maresh CM, Kraemer WJ (1996). Relationship between athletic performance tests and playing time in elite college basketball players. J. Strength Cond. Res..

[44] Pauole K, Madole K, Garhammer J, Lacourse M, Rozenek R (2000). Reliability and validity of the T-test as a measure of agility, leg power, and leg speed in college-aged men and women. J. Strength Cond. Res..

[45] Tobin DP, Delahunt E (2014). The acute effect of a plyometric stimulus on jump performance in professional rugby players. J. Strength Cond. Res..

[46] Requena B, González-Badillo JJ, Saez De Villareal ES, Ereline J, García I, Gapeyeva H (2009). Functional performance, maximal strength, and power characteristics in isometric and dynamic actions of lower extremities in soccer players. J. Strength Cond. Res..

[47] Esformes JI, Cameron N, Bampouras TM (2010). Postactivation potentiation following different modes of exercise. J. Strength Cond. Res..

[48] Till KA, Cooke C (2009). The effects of postactivation potentiation on sprint and jump performance of male academy soccer players. J. Strength Cond. Res..

[49] Wu C-C, Wang M-H, Chang C-Y, Hung M-H, Wang H-H, Chen K-C (2021). The acute effects of whole body vibration stimulus warm-up on skill-related physical capabilities in volleyball players. Sci. Rep..

[50] Naclerio F, Faigenbaum AD, Larumbe-Zabala E, Ratamess NA, Kang J, Friedman P (2014). Effectiveness of different postactivation potentiation protocols with and without whole body vibration on jumping performance in college athletes. J. Strength Cond. Res..

[51] Cormie P, Deane RS, Triplett NT, McBride JM (2006). Acute effects of whole-body vibration on muscle activity, strength, and power. J. Strength Cond. Res..

[52] Rittweger J, Beller G, Felsenberg D (2000). Acute physiological effects of exhaustive whole-body vibration exercise in man. Clin. Physiol..

[53] Bullock N, Martin DT, Ross A, Rosemond CD, Jordan MJ, Marino FE (2008). Acute effect of whole-body vibration on sprint and jumping performance in elite skeleton athletes. J. Strength Cond. Res..

[54] Kinser AM, Ramsey MW, O’Bryant HS, Ayres CA, Sands WA, Stone MH (2008). Vibration and stretching effects on flexibility and explosive strength in young gymnasts. Med. Sci. Sports Exer..

[55] Turner AP, Sanderson MF, Attwood LA (2011). The acute effect of different frequencies of whole-body vibration on countermovement jump performance. J. Strength Cond. Res..

[56] Duthie GM, Pyne DB, Ross AA, Livingstone SG, Hooper SL (2006). The reliability of ten-meter sprint time using different starting techniques. J. Strength Cond. Res..

[57] Mccurdy KW, Walker JL, Langford GA, Kutz MR, Guerrero JM, Mcmillan J (2010). The relationship between kinematic determinants of jump and sprint performance in division I women soccer players. J. Strength Cond. Res..

[58] Kavanaugh A, Ramsey MW, Sands WA, Haff GG, Stone MH (2011). Acute whole-body vibration does not affect static jump performance. Eur. J. Sport Sci..

[59] Young WB, McDowell MH, Scarlett BJ (2001). Specificity of sprint and agility training methods. J. Strength Cond. Res..

[60] Gullich A, Sehmidtbleicher D (1996). MVC-induced short-term potentiation of explosive force. New Stud. Athl..

[61] Gourgoulis V, Aggeloussis N, Kasimatis P, Mavromatis G, Garas A (2003). Effect of a submaximal half-squats warm-up program on vertical jumping ability. J. Strength Cond. Res..

[62] Chiu LZF, Fry AC, Weiss LW, Schilling BK, Brown LE, Smith SL (2003). Postactivation potentiation response in athletic and recreationally trained individuals. J. Strength Cond. Res..

[63] Pienaar C (2013). The acute effect of whole body vibration (WBV) training on power-related measurements of field hockey players. Afr. J. Phys. Health Educ. Recreat. Dance.

[64] Torvinen S, Sievänen H, Järvinen TAH, Pasanen M, Kontulainen S, Kannus P (2002). Effect of 4-min vertical whole body vibration on muscle performance and body balance: A randomized cross-over study. Int. J. Sports Med..

